# Jasmonic acid pretreatment improves salt tolerance of wheat by regulating hormones biosynthesis and antioxidant capacity

**DOI:** 10.3389/fpls.2022.968477

**Published:** 2022-07-22

**Authors:** Mo Zhu, Yan Liu, Pengkun Cai, Xiao Duan, Shifei Sang, Zongbo Qiu

**Affiliations:** ^1^College of Life Science, Henan Normal University, Xinxiang, China; ^2^Henan International Joint Laboratory of Agricultural Microbial Ecology and Technology, Henan Normal University, Xinxiang, China

**Keywords:** jasmonic acid, salt stress, wheat, transcriptome, antioxidative defense system

## Abstract

Salt stress is a severe environmental factor that detrimentally affects wheat growth and production worldwide. Previous studies illustrate that exogenous jasmonic acid (JA) significantly improved salt tolerance in plants. However, little is known about the underlying molecular mechanisms of JA induced physiochemical changes in wheat seedlings under salt stress conditions. In this study, biophysiochemical and transcriptome analysis was conducted to explore the mechanisms of exogenous JA induced salt tolerance in wheat. Exogenous JA increased salt tolerance of wheat seedlings by alleviating membrane lipid oxidation, improving root morphology, enhancing the contents of ABA, JA and SA and increasing relative water content. In the RNA-seq profiles, we identified a total of 54,263 unigenes and 1,407 unigenes showed differentially expressed patterns in JA pretreated wheat seedlings exposed to salt stress comparing to those with salt stress alone. Subsequently, gene ontology (GO) and KEGG pathway enrichment analysis characterized that DEGs involved in linoleic acid metabolism and plant hormone signal transduction pathways were up-regulated predominantly in JA pretreated wheat seedlings exposed to salt stress. We noticed that genes that involved in antioxidative defense system and that encoding transcription factors were mainly up- or down-regulated. Moreover, SOD, POD, CAT and APX activities were increased in JA pretreated wheat seedlings exposed to salt stress, which is in accordance with the transcript profiles of the relevant genes. Taken together, our results demonstrate that the genes and enzymes involved in physiological and biochemical processes of antioxidant system, plant hormones and transcriptional regulation contributed to JA-mediated enhancement of salt tolerance in wheat. These findings will facilitate the elucidation of the potential molecular mechanisms associated with JA-dependent amelioration of salt stress in wheat and lay theoretical foundations for future studies concerning the improvement of plant tolerance to abiotic environmental stresses.

## Introduction

Wheat (*Triticum aestivum* L.) is one of the most important cereal crops in the world in terms of its key contribution to food security (Ma et al., 2017). However, its growth, yield and quality are significantly affected by many abiotic stresses, such as drought, freezing, salinity and high and low temperatures. Furthermore, salt stress is likely to become more frequent and serious in the coming decades because of global climate deterioration, and thus turns into a serious threat to crop productivity in many parts of the world (Fahad et al., 2014; Formentin et al., 2018). Therefore, the efforts to elucidate the molecular mechanisms of crops that are responsible for salt tolerance have become increasingly imperative to crop breeding and cultivation.

Jasmonic acid (JA), a naturally occurring plant growth regulator, has been well-recognized to influence various physiological and biochemical functions in plants under unfavorable environmental conditions (Ahmad et al., 2017; Wang et al., 2021). Most importantly, emerging studies have showed that JA also plays a positive role in alleviating the negative impacts of various abiotic stresses on plants (Fahad et al., 2016; Ghassemi-Golezani, 2018; Sheteiwy et al., 2021). Ahmad et al. (2017) studied the effects of JA on faba bean under cadmium (Cd) stress and found that exogenous 100 μM JA mitigated the negative effects of Cd stress by reducing levels of hydrogen peroxide (H_2_O_2_), malondialdehyde (MDA) and Cd accumulation, and by improving proline content and antioxidant enzyme activities in plant leaves. Foliar application of 60 μM JA was shown to protect soybean (*Glycine max* L.) seedlings from salt stress mediated oxidative damage possibly through reducing lipid peroxidation, improving growth performance and modulating antioxidant defense and stomatal closure (Sheteiwy et al., 2021). In previous investigations, we found that exogenous JA effectively protected wheat seedlings from salt stress through decreasing oxidative damage and enhancing antioxidant capacity (Qiu et al., 2014). Although much attention has long been paid to elucidate the influences of JA on plant growth and developmental processes at morphological, physiological and biochemical levels, such as biomass yield, relative water contents, pigment contents, antioxidant compound production and antioxidant enzymatic activities (Qiu et al., 2014; Ahmad et al., 2017; Sheteiwy et al., 2021), there are very few studies concerning the impacts of JA on wheat growth under salt stress at molecular mechanisms, such as gene transcription and regulation.

RNA-Seq, a high-throughput sequencing technology, is the most effective way to characterize gene transcriptional changes and thus provides insights into the complex molecular mechanisms underlying plant response to various abiotic stresses. Recently, large-scale transcriptomic analysis has been widely used on various crops based on RNA-Seq to examine expression levels of each gene transcripts in response to cold stress (Wang et al., 2015), drought stress (Qiu et al., 2017) and heat stress (Xu and Huang, 2018). In a previous study, the global investigation of differentially expressed genes in the root and leaf tissues of wild rice under salt stress provided the gene ontology, metabolic pathways and unveiled the underlying molecular mechanisms of salt-stressed responses (Zhou et al., 2016)). Furthermore, Yao et al. (2018) provided an overview of the transcriptome of *Halogeton glomeratus* roots using high-throughput sequencing under 60 mM NaCl stress for 24 h. Although much progress has been achieved in deciphering the adaptive mechanisms of plants to environmental stresses, the possible mechanisms underlying salt tolerance conferred by JA in wheat are still unclear. Therefore, this present study was undertaken to investigate JA mediated regulation of gene expressions and metabolic pathways in wheat with respect to salt stress using the transcriptome sequencing technology, and to explore the molecular mechanisms of salt tolerance induced by exogenous JA in wheat. Our results provide deeper insights into the potential modulating mechanisms underlying the JA mediated enhancement of salt stress tolerance in wheat, and lay a foundation for future studies regarding the improvement of plant tolerance to environmental stresses.

## Materials and methods

### Plant materials, growth conditions, and treatments

Surface-sterilized wheat seeds (cv. Zhengmai No. 366, a winter wheat cultivar with high-yielding and multiple disease resistant) were germinated in the dark at 25°C for 48 h before being transplanted into Petri plates (diameter 16 cm) with 1/2 Hoagland solution for subsequent growth. The components of 1/2 Hoagland solution were as follows: MgSO_4_·7H_2_O (1 mM), KNO_3_ (2.5 mM), Ca(NO3)_2_·4H_2_O (2.5 mM), H_3_BO_3_ (47 μM), KH_2_PO_4_ (0.5 mM), EDTA-Fe (50 μM), MnCl_2_·4H_2_O (1 μM), CuSO_4_·5H_2_O (0.25 μM), ZnSO_4_·7H_2_O (1 μM), Na_2_MoO_4_·2H_2_O (0.1 μM). The seedlings were grown in a growth chamber with 25°C/16°C (day/night), illumination cycle 12/12 h at 750 μmol m^−2^ s^−1^ and relative humidity of 70 ± 5% (Zhu et al., 2022a). At the 1–2 leaf stage (8 days after germination), the seedlings were divided into the following four groups: (1) seedlings were sprayed with distilled water (the control, CK), (2) seedlings were sprayed with 100 μM JA solution (JA), (3) seedlings were treated with 150 mM NaCl solution (NaCl), and (4) seedlings were sprayed with 100 μM JA before additional 150 mM NaCl solution (JA+NaCl). For JA treatment, 100 μM JA was sprayed two times at 2-day intervals and control plants received the same volume of distilled water. Four days after commencing the JA treatments, 150 mM NaCl was added to the 1/2 Hoagland solution for seedlings with or without JA treatment. Hundred micrometer JA and 150 mM NaCl were chosen based on our previous studies (Qiu et al., 2014). Every treatment consisted of three petri plates with 100 seedlings each, and three replications were performed. At day 3 of salt stress, leaves and roots were collected respectively, immediately frozen in liquid nitrogen and stored at −80°C until further use. Then, the shoots and roots of another wheat seedlings were separated and each shoot and root of 12 randomly selected plants per treatment were weighed to determine the fresh weight. Subsequently, shoots and roots were placed in an oven at 65°C to reach a constant weight and then dry weight of each sample was measured.

### Determination of MDA and relative water content (RWC)

According to a previously described method (Qiu et al., 2016), MDA content in roots was determined using the 2-thiobarbituric acid method. Relative water content (RWC) in fresh leaves was carried out using the method described in Barrs and Weatherley (1962).

### Assay of root morphology

The roots of wheat seedlings (15 plants per treatment) were collected and washed in distilled water. Next, the fresh roots were scanned using a WinRHIZO root analysis system (LA6400XL, Regent Instruments Inc., Canada), and then resulting root images were used to determine root morphology (root length, root diameter, root surface area and number of root tips) according to the method described by Zhu et al. (2022b).

### RNA isolation, library construction and sequencing

Total RNA was extracted from frozen roots in different treatments with Trizol reagent (Invitrogen, USA) as suggested by the manufacturer. The concentration and quality of RNA samples were assessed using an Agilent 2100 Bioanalyzer and only RNA samples that met an RNA integrity number above 8 were used for sequencing. RNA-Seq libraries were constructed using NEBNext Ultra RNA Directional Library Prep Kit for Illumina (NEB, USA) as described by Liu et al. (2018) and finally, were sequenced on an Illumina Hiseq™ 4000 platform at Novogene Bioinformatics Technology Co. Ltd (Beijing, China). All raw sequence data are deposited in the NCBI Sequence Read Archive (SRA) database under the accession number PRJNA513931.

### Transcriptome assembly and differentially expressed genes (DEGs) identification

Clean reads were obtained by filtering out adapter sequences, poly-N, unknown nucleotides and low-quality reads with scores of <20 at the 3′ and 5′ ends from the raw data. All downstream analyses were based on clean reads with high quality. Transcriptome assembly was accomplished by Trinity software with the default parameters (Grabherr et al., 2011). RPKM (reads per kilobase of coding sequence per million reads) was used to quantify the mapped gene expression levels. DESeq R package was used to assessed differentially expressed genes (DEGs) among the four treatments based on the following thresholds: |log_2_ (fold change)| ≥ 2 and false discovery rate (FDR) corrected *p*-value < 0.05 (Benjamini and Yekutieli, 2001). All identified DEGs were analyzed *via* sequence alignment with the Gene Ontology (GO) and Kyoto Encyclopedia of Genes and Genomes (KEGG) databases using Blast2GO (Conesa et al., 2005).

### Assessment of antioxidant enzymatic activity

To measure enzyme activities, 1.0 g fresh roots were ground under ice-cold conditions in a 3 mL 50 mM phosphate buffer (pH 7.8) containing 1 mM DTT, 1 mM EDTA and 2% PVP. After centrifugation at 12 000 g for 20 min at 4°C, the supernatant was used to examine the activity of peroxidase (POD) and catalase (CAT) following the method of Zhang and Kirkham (1994), that of ascorbate peroxidase (APX) based on the decrease in ascorbate (Nakano and Asada, 1981), that of superoxide dismutase (SOD) using nitro blue tetrazolium as described by Giannopolitis and Ries (1977).

### Phytohormones quantification

Salicylic acid (SA), jasmonic acid (JA) and abscisic acid (ABA) were extracted and quantified from the roots of wheat seedlings according to Réthoré et al. (2020) with some modification. SA, JA and ABA were detected, identified and analyzed by a high-performance liquid chromatography combined with electrospray ionization tandem mass spectrometry (HPLC-ESI-MS/MS) system. Roots were ground to a fine power in liquid nitrogen using a mortar and pestle, respectively. Then the fine powder of each sample (1 g) was added 10 ml precooled extraction solution containing 95% isopropanol and 5% hydrochloric acid shaken on a shaking bed overnight at 4°C. Then, the extraction solution was added 20 ml CH_2_Cl_2_ following shaken at 800 rpm for 1 h at 4°C. After centrifuging (13 000 rpm, 4°C, 10 min), low organic layer was collected and blow-dried with nitrogen gas at room temperature, and then dissolved in 500 μl methanol and passed through a 0.22 μm microporous membrane before injection into the system. Samples (5 μl) were separated and analyzed by HPLC-ESI-MS/MS using an Agilent 1290 HPLC coupled to a SCIEX 6500 Q-TRAP (Applied Biosystems, California, USA). The HPLC-ESI-MS/MS conditions used for the analysis and quantification of SA, JA and ABA are provided in Supplementary Table 1.

### RT-qPCR validation and expression analysis

Quantitative real-time PCR (RT-qPCR) assay was conducted to confirm and analyze expression levels of twelve candidate genes and gene-specific primers used are summarized in Supplementary Table 2. First-Strand cDNA was synthesized from 2 μg total RNA of flash-frozen fresh roots according to the directions of the reverse transcription kit (TaKaRa, Dalian, China). RT-qPCR was performed as previously described (Zhu et al., 2022c). Each reaction had three biological replicates and *tubulin* (DQ435660.1) was used as a reference gene to normalize the expression of the candidate genes using the 2^−ΔΔCt^ method (Livak and Schmittgen, 2001).

### Statistical analysis

The data for plant growth, root morphology, antioxidant enzymes, gene expression and hormones levels were analyzed by SPSS (version 21.0). Duncan's multiple range test through one-way analysis of variance (ANOVA) was used to evaluate significant difference among samples (*p* < 0.05).

## Results

### Exogenous jasmonic acid affects growth and MDA content in wheat seedlings under salt stress

**Figure 1:**
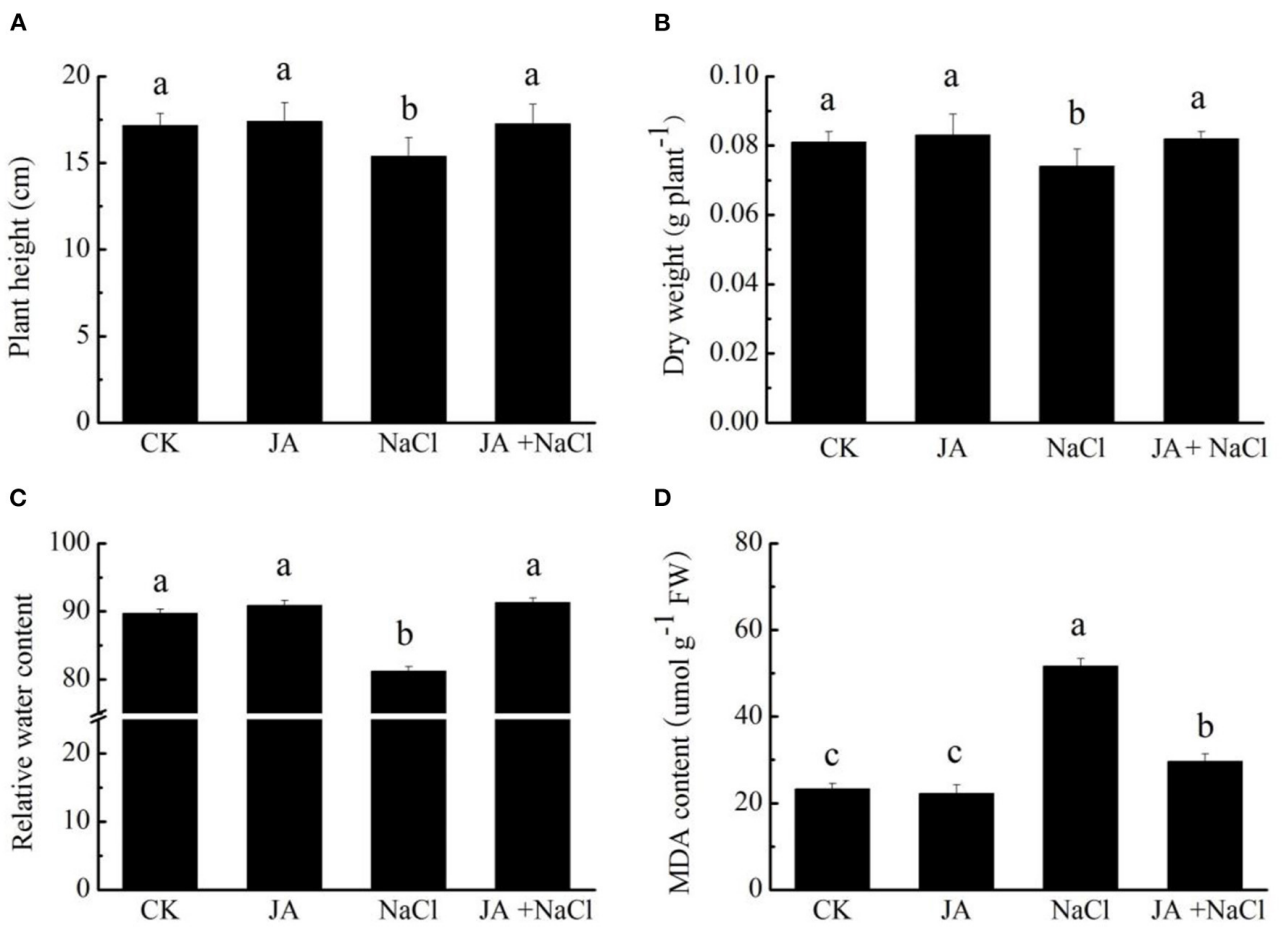
Effects of exogenous jasmonic acid (JA) on **(A)** plant height, **(B)** dry weight, **(C)** relative water content (RWC) and **(D)** MDA content in wheat seedlings under salt stress. CK, control group; NaCl, salt stress; JA, 100 μM JA alone; JA + NaCl, salt stress combined with 100 μM JA. The bars (means ± SD, *n* = 6) labeled with different letters indicate significant differences (*p* < 0.05) between treatments according to Duncan's multiple range test.

To assess the roles of exogenous JA on wheat, plant height, dry weight, relative water content (RWC) and MDA content were measured in wheat seedlings treated with or without exogenous JA under salt stress condition. Salt treatment significantly reduced plant height, dry weight and relative water content by 10.4, 8.6, and 9.5%, respectively, comparing to controls (Figures 1A–C). However, the application of exogenous JA significantly increased plant height by 12.2%, dry weight by 10.8% and relative water content by 12.4% over the values of salt-treated wheat seedlings. In addition, salt stress dramatically increased MDA content by 121.3% in wheat seedlings comparing to controls. However, JA application markedly decreased MDA content in wheat by 42.7% in comparison to the salt treated plants (Figure 1D). Application of JA alone did not notably influence plant height, dry weight, relative water content and MDA content in wheat comparing to those of controls.

### Exogenous jasmonic acid influences root phenotype and morphological characteristics of wheat seedlings under salt stress

**Figure 2:**
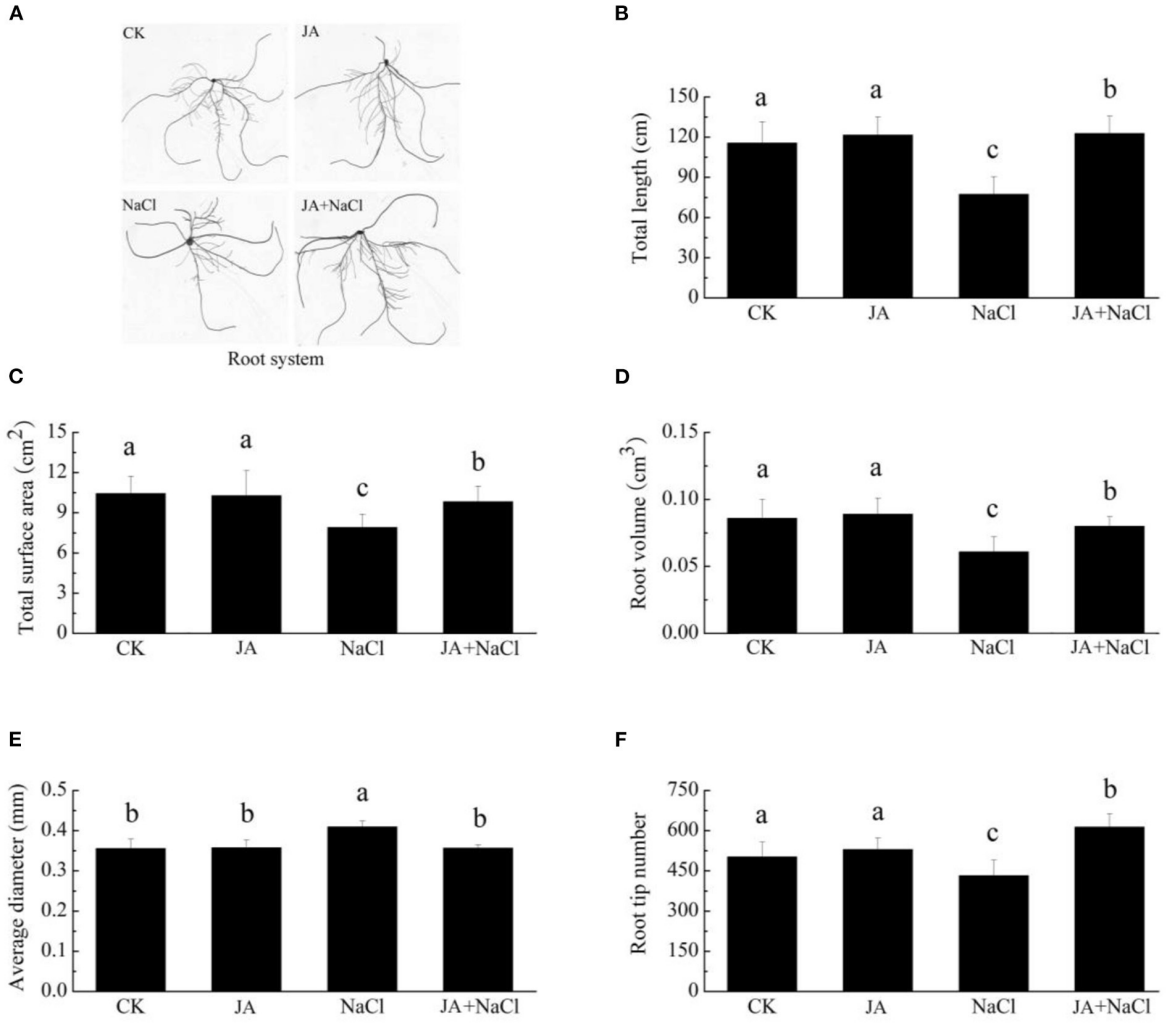
Effects of exogenous jasmonic acid on root phenotype and morphology in wheat seedlings under salt stress. See notes to Figure 1. **(A)** Root system, **(B)** Total root length, **(C)** Total root surface area, **(D)** Root volume, **(E)** Average root diameter, and **(F)** Root tip numbers. The bars (means ± SD, *n* = 6) labeled with different letters indicate significant differences (*p* < 0.05) between treatments according to Duncan's multiple range test.

Roots of wheat seedlings under different treatments showed obvious morphological differences (Figure 2A). As compared to control group, salt stress exhibited substantial reduction in total root length by 33.13%, total root surface area by 24.2%, root volume by 29.1%, root tip numbers by 13.9%, whereas increase in average root diameter by 15.2%. However, exogenous JA treatment with salt stress was found to be effective in improving root morphology and enhanced total root length by 58.8%, total surface area by 24.2%, root volume by 31.3% and root tip numbers by 41.8% with respect to plants treated with salt stress alone (Figures 2B–F). In addition, JA treatment alone did not affect root length, diameter, volume, surface area and tips number in comparison to those of controls.

### Quantitative assessment of transcriptome data

In order to gain more insights into the molecular mechanisms underlying JA-mediated enhancement of salt tolerance in wheat seedlings, four libraries (three replicates per library) of wheat treated with or without JA under control or salt stress conditions were constructed. Each library consisted of more than 6.1 Gb of clean bases with a Q20 percentage over 97%, Q30 percentage over 93%, and a GC percentage between 54.23 and 56.21% (Table 1). All clean reads from the 12 sequencing libraries were used to construct the transcriptome based on Trinity software. In total, 142,240 transcripts were obtained. The median-, shortest- and longest length for these transcripts was 470 bp, 201 bp and 10,795 bp, respectively. The transcripts were further clustered into 54,263 unigenes with a mean length of 930 bp (N50 = 985 bp; Supplementary Table 3). To obtain comprehensive information of gene functions, 54,263 unigenes were blasted against Nr (NCBI non-redundant protein sequences), Pfam (protein family), Swiss-Prot (a manually annotated and reviewed protein sequences), GO (Gene Ontology), COG (Cluster of Orthologous Groups of proteins) and KEGG (Kyoto Encyclopedia of Genes and Genomes) databases. Of these unigenes, 24,388 (44.94%) had significant matches in the Nr database. The numbers of sequences that were highly similar to entries in the GO, KEGG, Pfam, Swiss-Prot and COG databases were 16,596 (30.58%), 9,124 (16.81%), 15,874 (29.25%), 13,811 (25.45%), and 21,403 (39.44%), respectively (Supplementary Table 4).

**Table 1:** Summary of sequence assembly after Illumina sequencing.

**Sample**	**Raw reads**	**Clean reads**	**Clean bases**	**Q20 (%)**	**Q30 (%)**	**GC content (%)**
CK_1	43,358,966	41,645,954	6.13G	98.03	94.52	56.11
CK_2	36,756,718	35,593,966	5.24G	98.44	95.53	56.21
CK_3	41,287,565	39,620,148	5.83G	98.12	94.75	56.02
JA_1	38,542,044	36,055,850	5.30G	98.01	94.48	54.93
JA_2	47,240,416	44,878,396	6.57G	97.61	93.82	54.85
JA_3	43,215,064	40,958,731	5.86G	97.82	94.21	54.32
NaCl_1	40,796,460	39,546,508	5.82G	98.02	94.51	54.23
NaCl_2	44,781,028	43,114,712	6.31G	97.64	93.79	54.37
NaCl_3	42,315,607	40,921,473	6.05G	97.86	94.23	54.64
JA+NaCl_1	40,502,286	38,959,102	5.72G	97.88	94.26	54.61
JA+NaCl_2	51,309,000	48,678,204	7.10G	97.37	93.23	54.88
JA+NaCl_3	43,286,918	41,847,269	6.19G	97.67	94.21	54.87

*CK_1, CK_2 and CK_3: Control sample (three independent biological replicates)*.

*JA_1, JA_2 and JA_3: 100 μM JA treated sample (three independent biological replicates)*.

*NaCl_1, NaCl_2 and NaCl_3: 150 mM NaCl treated sample (three independent biological replicates)*.

*JA+NaCl_1, JA+NaCl_2 and JA+NaCl_3: 100 μM JA +150 mM NaCl treated sample (three independent biological replicates)*.

*Q20: The percentage of bases with a Phred value >20*.

*Q30: The percentage of bases with a Phred value >30*.

### Differentially expressed genes in wheat

**Figure 3:**
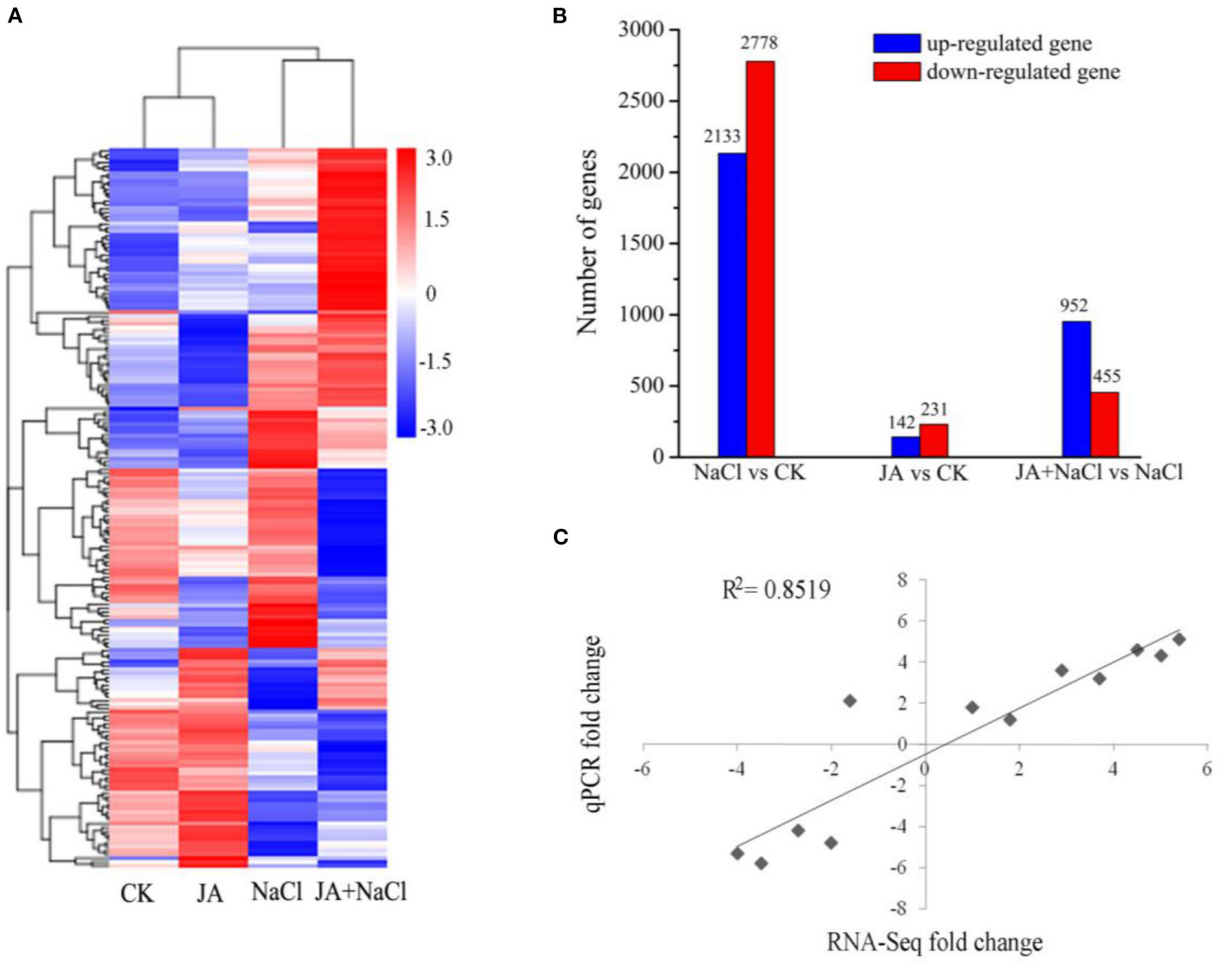
Effects of exogenous jasmonic acid on differentially expressed genes in wheat seedlings with or without JA treatment under salt stress. **(A)** Hierarchical clustering of all the DEGs based on log10 RPKM (number of reads per kilobase per million clean reads) values. The color (from blue to red) represents gene expression level from low to high. **(B)** The up-regulated and down-regulated number of DEGs in NaCl vs. CK, JA vs. CK, and JA + NaCl vs. NaCl comparisons. The numbers of DEGs are shown in the diagram. **(C)** Correlation of RNA-seq (y axis) and quantitative real-time PCR (qPCR) expression data (x axis). CK, control group; NaCl, alone salt stress; JA, 100 μM JA alone; JA + NaCl, salt stress combined with 100 μM JA.

In order to gain a global view of transcript expression in JA treated wheat seedlings under salt stress, a heat-map was constructed by clustering of all the DEGs to observe the gene expression level (Figure 3A). Compared with controls (CK), there were 4,911 DEGs in NaCl treatment (NaCl vs. CK comparison), including 2,133 up-regulated and 2,778 down-regulated genes and there were 373 DEGs in JA treatment (JA vs. CK comparison), including 142 up-regulated and 231 down-regulated genes (Figure 3B). However, comparing between JA+NaCl and NaCl treatments, 1,407 DEGs were identified, of which 952 were up-regulated and 455 were down-regulated (Figure 3B). The 1,407 DEGs that were associated with the response to exogenous JA were thus considered important candidates for further investigation.

To confirm the expression level of DEGs from RNA-seq profiles, we randomly selected 12 DEGs for quantitative real-time PCR (RT-qPCR) in JA-mediated salt tolerance. The results of RT-qPCR showed a significant positive correlation with the RNA-seq data (*R*^2^ = 0.8519) (Figure 3C), indicating the reliability of RNA-Seq data in this study.

GO enrichment analysis was further evaluated to reveal gene functions of DEGs. In total, the 1,407 DEGs in the comparison of JA+NaCl and NaCl treatment were enriched into molecular function, cellular component and biological process. Among these groups, the terms “plasma membrane,” “protein serine/threonine kinase activity,” and “protein phosphorylation” are dominant in each of the three main categories (Supplementary Figure 1). We also conducted a Kyoto Encyclopedia of Genes and Genomes (KEGG) enrichment analysis of the DEGs to explore the main pathways active in JA induced salt tolerance in wheat. Between the NaCl and CK libraries, 886 DEGs were assigned to 20 KEGG pathways, including starch and sucrose metabolism (ko00500) with 119 genes (35 genes were upregulated and 84 genes were downregulated), plant hormone signal transduction (ko04075) with 111 genes (74 genes were upregulated and 37 genes were downregulated). Between JA+NaCl and NaCl treatment, 298 DEGs were assigned to 12 KEGG pathways, such as linoleic acid metabolism (ko00591) with three genes up-regulated and plant hormone signal transduction (ko04075) with 18 genes up-regulated (Supplementary Table 5).

### Jasmonic acid regulated genes involved in hormone biosynthesis in wheat seedlings under salt stress

**Figure 4:**
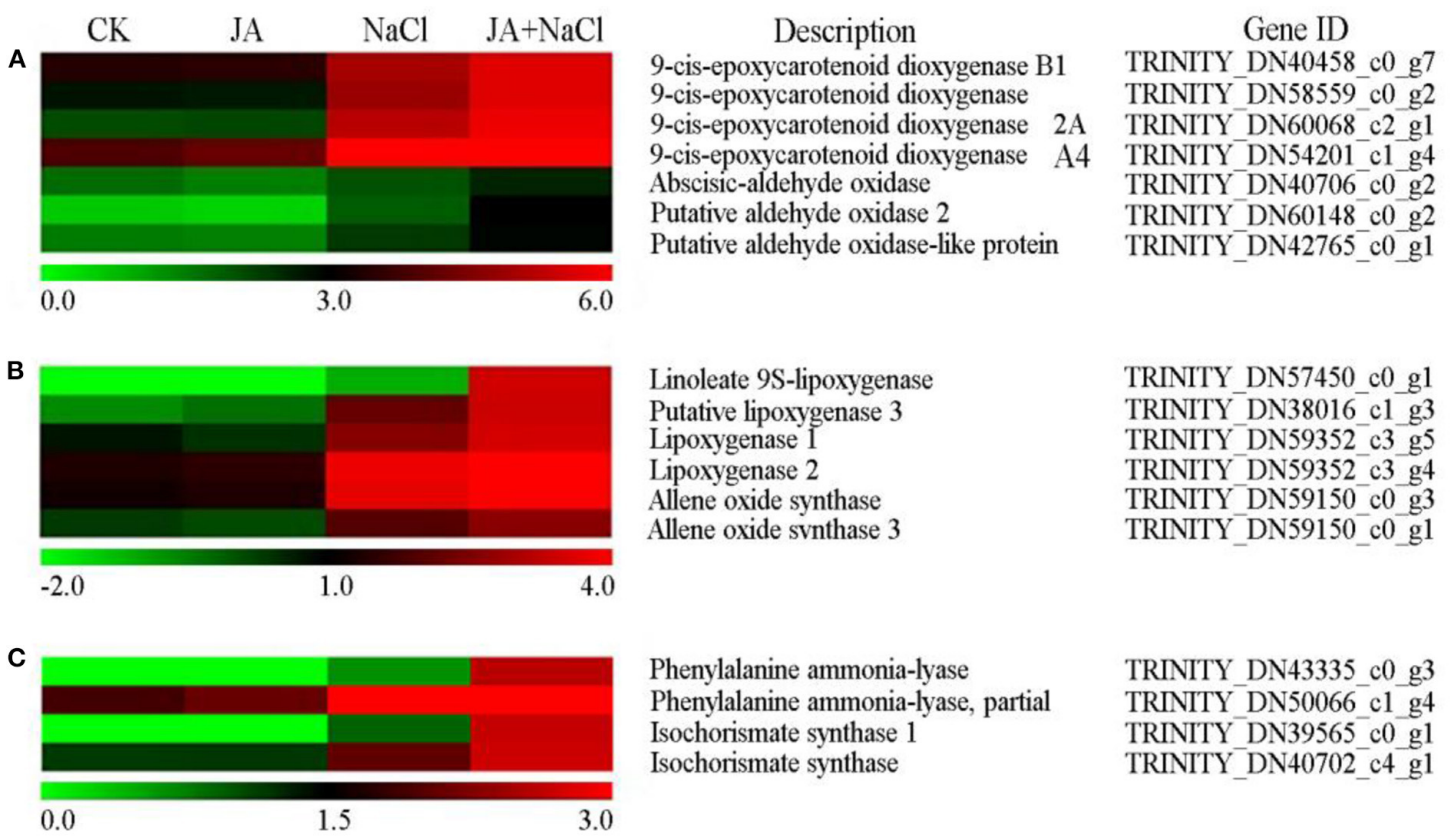
Effects of exogenous jasmonic acid on the expression of DEGs related to hormone biosynthesis in wheat seedlings under salt stress. Genes related to **(A)** abscisic acid, **(B)** jasmonic acid and **(C)** salicylic acid. The bars represent the scale of the expression levels for each gene as indicated by green (low expression) and red rectangles (high expression). CK, control group; NaCl, alone salt stress; JA, 100 μM JA alone; JA + NaCl, salt stress combined with 100 μM JA.

From the transcriptome analysis, it was found that exogenous JA regulated the expression of many DEGs involved in phytohormone biosynthesis, including abscisic acid, jasmonic acid and salicylic acid biosynthesis (Figure 4). The DEGs encoding 9-cis-epoxycarotenoid dioxygenase (NCED) (DN40458_c0_g7, DN58559_c0_g2, DN60068_c2_g1) and aldehyde oxidase 2 (DN60148_c0_g2 and DN42765_c0_g1) in ABA biosynthesis pathway were up-regulated in JA + NaCl vs NaCl comparison (Figure 4A). Compared with NaCl treatment, four genes in JA + NaCl treatment groups were significantly up-regulated, including DEGs encoding linoleate 9S-lipoxygenase (DN57450_c0_g1), lipoxygenase 3 (DN38016_c1_g3), lipoxygenase 1 (DN59352_c3_g5) and allene oxide synthase (DN59150_c0_g1) in JA biosynthesis pathway (Figure 4B). Additionally, three DEGs involved in SA biosynthesis were significantly up-regulated in JA + NaCl vs. NaCl comparison, including the gene (DN43335_c0_g3) encoding phenylalanine ammonia-lyase and two DEGs (DN39565_c0_g1 and DN40702_c4_g1) encoding isochorismate synthase (Figure 4C).

### Exogenous jasmonic acid treatment regulated hormone levels in wheat under salt stress

**Figure 5:**
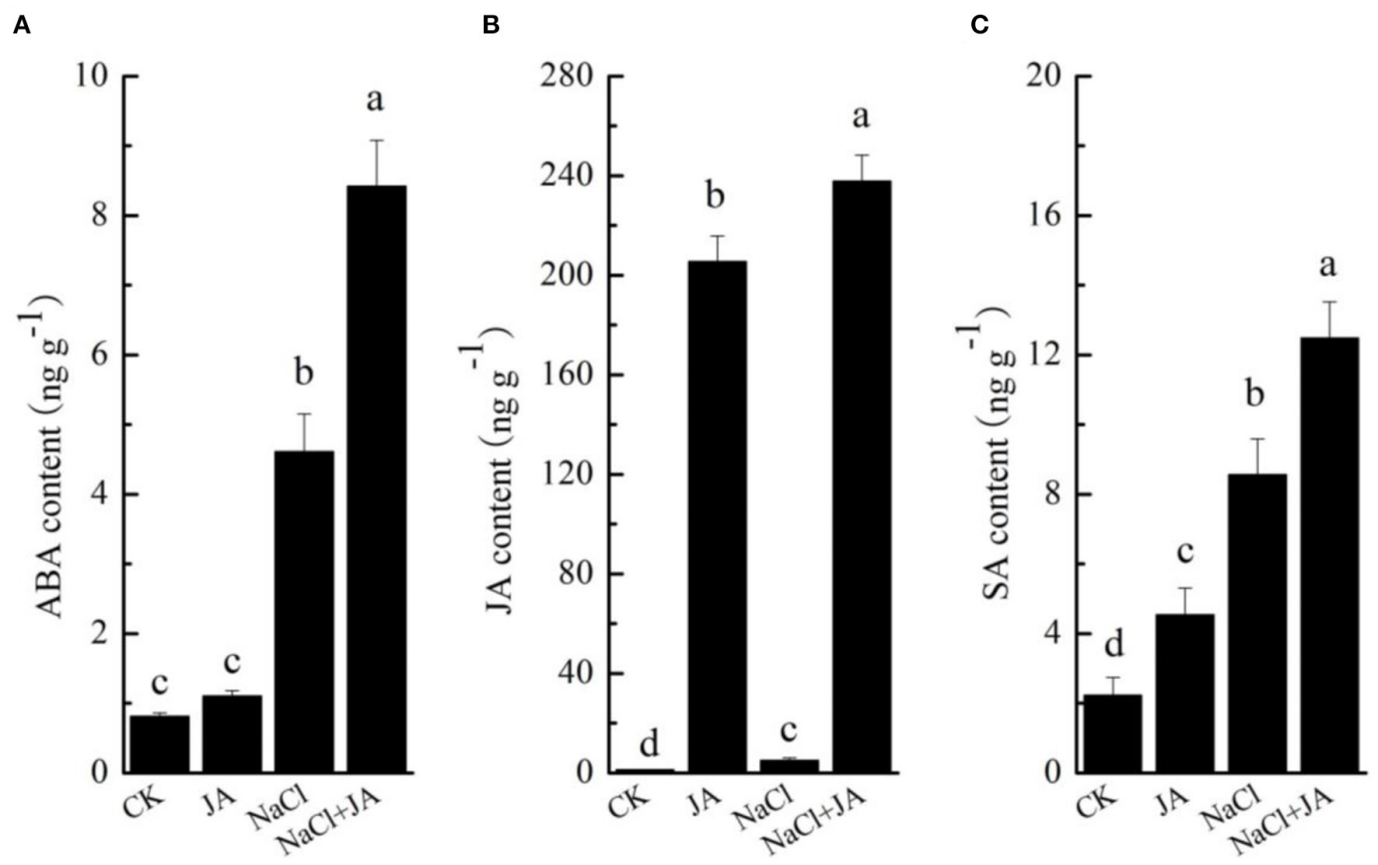
Effects of exogenous jasmonic acid on the contents of (**A**) abscisic acid (ABA), (**B**) jasmonic acid (JA) and (**C**) salicylic acid (SA) in wheat seedlings under salt stress. CK, control group; NaCl, alone salt stress; JA, 100 μM JA alone; JA + NaCl, salt stress combined with 100 μM JA. The bars (means ± SD, *n* = 3) labeled with different letters indicate significant differences (*p* < 0.05) between treatments according to Duncan's multiple range test through one-way analysis of variance (ANOVA).

As shown in Figure 5, the content of ABA, JA, and SA were significantly affected by both NaCl stress and exogenous JA application. NaCl stress triggered a significant increase in ABA, JA, and SA concentrations by 469, 282, and 283%, respectively, However, exogenous JA application further enhanced the contents of ABA, JA, and SA by 83, 4,547, and 46% over the corresponding contents in wheat with NaCl treatment. In addition, treatment of JA alone did not significantly affect the ABA content in comparison to the control.

### JA mediated redox-related gene expressions and antioxidant enzyme activities in wheat seedlings under salt stress

**Figure 6:**
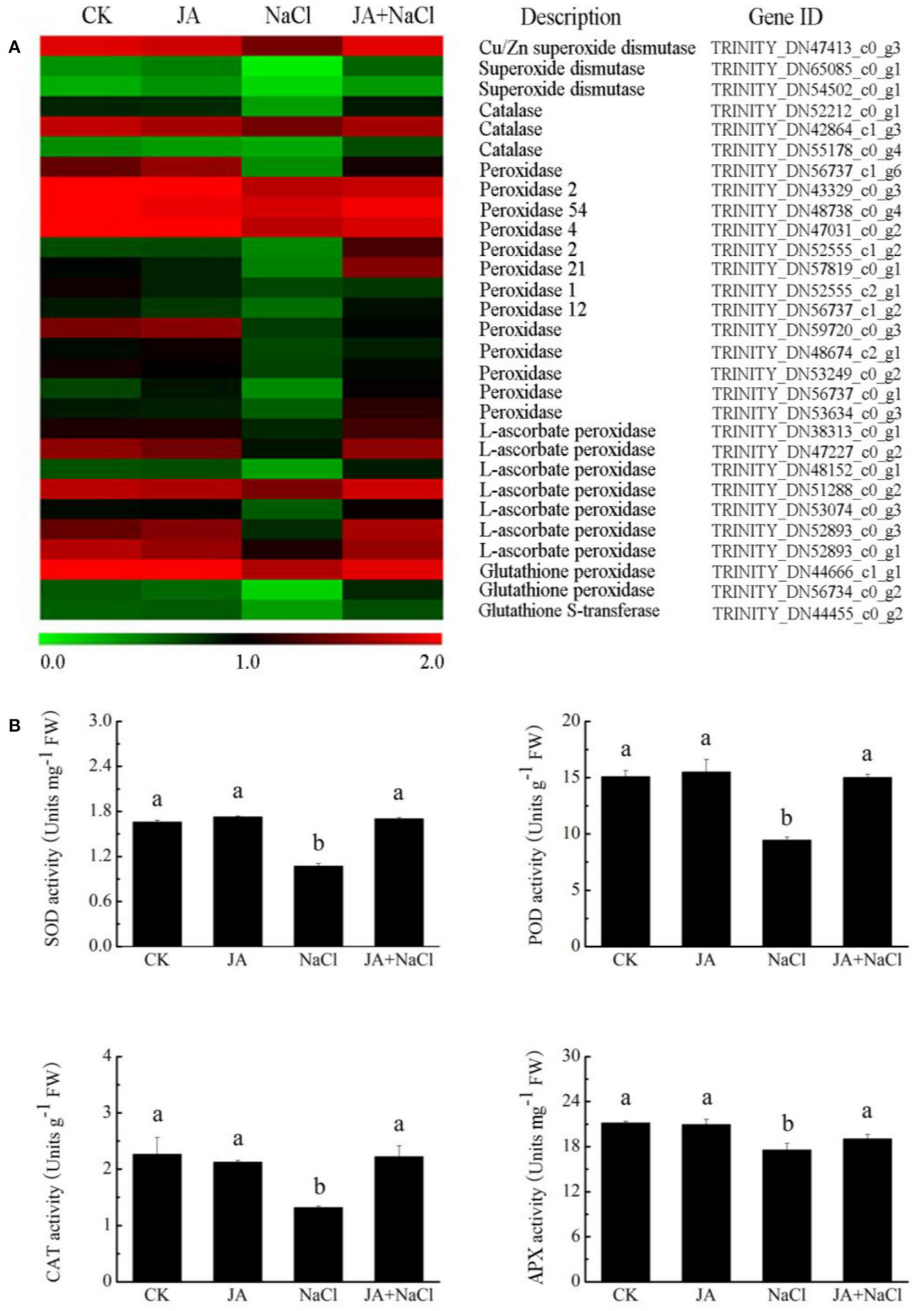
Effects of exogenous jasmonic acid on redox-related gene expression and antioxidant enzyme activities in wheat seedlings under salt stress. **(A)** Heatmap of DEG expressions. The bar represents the scale of the expression level for each gene as indicated by green (low expression) and red rectangles (high expression). **(B)** Activities of SOD, POD, CAT and APX. CK, control group; NaCl, alone salt stress; JA, 100 μM JA alone; JA + NaCl, salt stress combined with 100 μM JA. The bars (means ± SD, *n* = 6) labeled with different letters indicate significant differences (*p* < 0.05) between treatments according to Duncan's multiple range test.

RNA-Seq analysis showed that the genes related to *SOD, APX, CAT*, and *POD* were notably regulated in wheat plants with or without JA treatment under salt stress condition. As compared to control plants, thirteen antioxidant enzyme genes, including encoding one *CAT* (DN52212_c0_g1), one *SOD* (DN47413_c0_g3), six *POD* (DN56737_c0_g1, DN57819_c0_g1, DN52555_c2_g1, DN56737_c1_g1, DN59720_c0_g3, DN53634_c0_g3), and five *APX* (DN38313_c0_g1, DN47227_c0_g2, DN53074_c0_g3, DN52893_c0_g3, DN52893_c0_g1) were significantly down-regulated in the NaCl treated plants (Figure 6A). Compared with NaCl treatment, fifteen genes in JA + NaCl treated plants were significantly up-regulated, including the genes encoding *SOD, CAT, POD, APX* and *GST*.

To further confirm the relationship between antioxidant enzymes and salt tolerance in wheat, the enzyme activities in wheat plants with or without JA treatment under salt stress condition were measured. When compared with those in control plants, the activities of SOD, APX, CAT and POD in NaCl-treated wheat decreased by 35.3, 17, 41.7, and 37.4%, respectively. However, in JA-treated plants under salt stress, the activities of SOD, APX, CAT and POD notably increased by 58.6, 8.5,68.4, 59%, respectively as compared to those in NaCl-treated wheat (Figure 6B). There is no dramatical difference in the activities of the enzymes between controls and JA-treated plants.

### JA regulated the expression of transcription factor coding genes in wheat seedlings under salt stress

**Figure 7:**
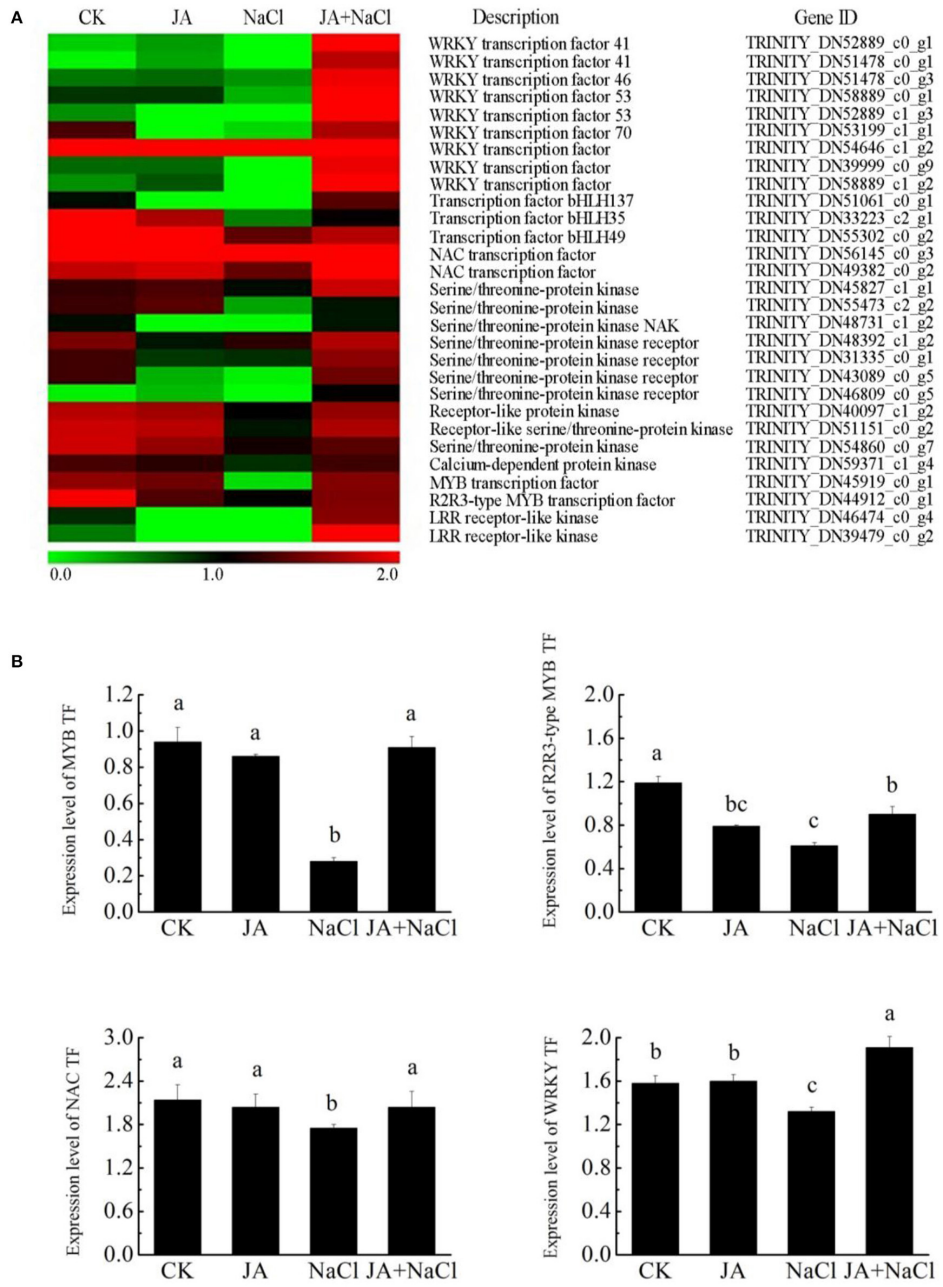
Effects of exogenous jasmonic acid on the expressions of DEGs related to transcription factor in wheat seedlings under salt stress. **(A)** Heatmap of DEG expressions. The bar represents the scale of the expression level for each gene as indicated by green (low expression) and red rectangles (high expression). **(B)** RT-qPCR analysis of the expression profiles of four transcripts. CK, control group; NaCl, alone salt stress; JA, 100 μM JA alone; JA + NaCl, salt stress combined with 100 μM JA. The bars (means ± SD, *n* = 6) labeled with different letters indicate significant differences (*p* < 0.05) between treatments according to Duncan's multiple range test.

Through the transcriptome analysis, we found that the transcripts encoding *MYB* (myeloblastosis related), *NAC* (NAM/ATAF1/CUC2), *R2R3-MYB, WRKY*, and *bHLH* (basic helix-loop-helix) were mostly downregulated under salt stress. However, most of them were upregulated in JA-treated plants under salt condition. In addition, JA treatment alone, in most cases, did not alter the expression level of *MYB, NAC, R2R3-MYB, bHLH* and *WRKY* in comparison to the control (Figure 7A).

We further examined the expression levels of four DEGs, *MYB, NAC, R2R3-MYB* and *WRKY*, by RT-qPCR in wheat seedlings with or without JA treatment under salt stress condition (Figure 7B). Our results showed that the expression levels of *MYB, NAC, R2R3-MYB* and *WRKY* were lower in NaCl treated plants. In contrast, significantly higher expression levels of *MYB, NAC, R2R3-MYB* and *WRKY* were also observed in JA-treated plants under salt stress. In addition, the expression trends of four genes were also basically consistent with the RNA-Seq data, although there were some differences in expression levels.

## Discussion

### Exogenous JA improves wheat seedlings growth and root morphology under salt stress

Salinity is known as one of the most deleterious abiotic stresses to plants by negatively affected growth, yield and productivity. The detrimental influences of salt stress on plant growth and biomass have been extensively reported in wheat (Qiu et al., 2014), faba bean (Nahhas et al., 2021), soybean (Sheteiwy et al., 2021) and *Brassica napus* (Huang et al., 2022). Our results further confirmed the negative impacts of salinity on wheat growth. It was found that salt stress obviously decreased plant height, dry weight and root morphology (Figures 1, 2). However, supplementation of exogenous JA significantly improved plant growth (plant height, dry weight) and root morphology (root length, volume, surface area and tips number) in wheat seedlings exposed to salt stress. Our results were in line with the study of Sheteiwy et al. (2021), who also demonstrated that foliar application with jasmonic acid significantly improved plant height, root length and the fresh and dry weight in soybean seedlings subjected to 100 mM NaCl stress. Exogenous JA was also reported to notably trigger plant growth in wheat under salt stress (Qiu et al., 2014), in *Brassica* under drought stress (Alam et al., 2014), in *Glycine max* under nickel toxicity (Sirhindi et al., 2016) and in faba bean under cadmium stress conditions (Ahmad et al., 2017). Therefore, JA treatment was regarded as a promising sustainable strategy for amending plant growth under stress conditions by improving wheat seedlings growth and root morphology and hence enhancing tolerance to salt stress. However, there is little information available in regard to the mechanisms of JA application in improving salt tolerance of wheat. To better understand the underlying biophysiochemical and molecular mechanisms, physiological and RNA-Seq analysis were conducted. It was clearly shown that growth, root morphology and transcriptome changes were mediated by JA-treated wheat seedlings under salt stress condition (Figures 1, 3).

### Exogenous jasmonic acid activated antioxidant defense system in wheat under salt stress

GO enrichment analysis showed that genes involved in the oxidation-reduction process and in response to oxidative stress were enriched in JA treated wheat seedlings under salt stress as compared to salt stress alone (Supplementary Figure 1). After salt stress, reactive oxygen species (ROS) are rapidly induced and accumulated and then putatively caused membrane and ion homeostasis disruption, resulting in oxidative destruction of amino acids and protein structures in plants (Qiu et al., 2014; Sheteiwy et al., 2021; Huang et al., 2022). In order to relieve the deleterious effects of salt stress, plants have developed a suite of antioxidative defense system which consists of ROS-scavenging enzymes, such as APX, SOD, CAT and anti-oxidative compounds, such as glutathione (GSH), ascorbic acid (AsA) and flavonoids to take over salt stress induced oxidative damages Sheteiwy et al., 2021; Shen et al., 2022. Higher levels of antioxidant enzymes and compounds in plants have been widely reported to be involved in improving tolerance against salt induced oxidative stress (Nahhas et al., 2021; Al-Zahrani et al., 2022). Furthermore, numerous studies have previously demonstrated that exogenous JA effectively reduced the accumulation of ROS under salt stress due to significant enhancement of antioxidant enzyme activities, including APX, POD, CAT, GR, and SOD thus improved stress tolerance in plants (Qiu et al., 2014; Sirhindi et al., 2016; Sheteiwy et al., 2021). Consistently, in our study, strongly up-regulated transcripts of genes encoding copper chaperone, glutathione S-transferase, plant peroxidase, ascorbate peroxidase, class III peroxidase and catalase were also modulated in JA treated wheat seedlings under salt stress as compared to salt stress alone, accompanied by significant increase in the activities of SOD, POD, APX and CAT (Figure 6). Similar results were obtained in *B. napus* where the combined application of exogenous MJ and As stress resulted in significant up-regulation of transcript levels of *SOD, POD, APX* and *CAT* genes that confer to As stress tolerance (Farooq et al., 2016). Using RT-qPCR, Sheteiwy et al. (2021) have also shown that foliar application with jasmonic acid enhanced antioxidant defense systems and improved salt stress tolerance in soybean by increasing the expression levels of *SOD, POD, CAT*, and *APX* which were in accordance with the increase of SOD, POD, CAT, and APX activities. Therefore, in agreement with previous studies, JA application was capable of effectively upregulating the transcript levels of *SOD, POD, APX* and *CAT*, which might contribute to the increase in the activities of SOD, POD, CAT and APX in JA treated wheat seedlings response to salt stress and thus resulting in the protection against oxidative damage induced by salt stress.

### Exogenous jasmonic acid regulates transcription factors involved in salt stress

Transcription factors are fundamental to the regulation of gene expression that was related to the response of diverse abiotic stresses and are responsible for transmitting stress signals and activating stress-responsive genes in plants (Hong et al., 2016; Wei et al., 2017). Recently, much attention has focused on numerous genes associated with stress responsive transcription factors and a large number of transcription factors belong to different families, such as *MYB, NAC, WRKY, AP2/ERF* (apetala ethylene responsive element binding protein), and *bHLH* (basic helix-loop-helix) have been identified to be involved in plant response to salt stress (Huang et al., 2015; Wei et al., 2017). It has been reported that *WRKY* transcription factors were involved in enhancing drought and salt stress tolerance in transgenic tobacco (Wang et al., 2013) and *Arabidopsis thaliana* (Ma et al., 2019) by regulating osmotic balance and reducing ROS accumulation. Increasing evidence had demonstrated that *NAC* transcription factors conferred abiotic stress adaptation in plants by modulating the antioxidant defense system that protects plants against oxidative damage (Mao et al., 2012; Hong et al., 2016). A recent study conducted by Xu et al. (2015) showed that *TaNAC29* conferred salt stress tolerance in wheat by reducing membrane damage and H_2_O_2_ accumulation, and regulating the antioxidant system that protects plants from oxidative damage. In addition, overexpression of *TaODORANT1*, a R2R3-type *MYB* transcription factor gene isolated from wheat, improved salt stress tolerance by upregulating the transcript levels of several ROS- and stress-related genes in transgenic tobacco (Wei et al., 2017). In this study, we found that 15 transcription factors including 5 *NAC*, 8 *WRKYs* and 2 *R2R3-MYB* were not induced by salt stress, but were significantly up-regulated in JA treated wheat seedlings under salt stress (Figure 7). These results are similar to the findings of Zhang et al. (2021), who analyzed the transcription factors induced by melatonin under salt stress, and found that a total of 98 transcription factor genes, including *AP2/ERF-ERF, WRKY, NAC* and *C2H2*, were up-regulated by melatonin under salt stress. In addition, the RT-qPCR analysis further indicated that the transcripts of *MYB, R2R3-MYB, WRKY*, and *NAC* domain transcription factor were significantly induced by JA in wheat seedlings exposed to salt stress. It was shown that over-expression of *NAC2* and *R2R3-type MYB* transcription factor genes in wheat enhanced multiple abiotic stress tolerances (Mao et al., 2012; Wei et al., 2017). As a result, the higher transcriptional levels of *NAC, WRKY*s, and *R2R3-MYB* transcription factor genes induced by JA were likely to contribute to the increase tolerance of wheat seedlings to salt stress.

### Exogenous jasmonic acid regulated genes involved in ABA, JA, and SA biosynthesis and contributed to salt stress tolerance in wheat

ABA is an essential and versatile plant hormone with sesquiterpene structure, where increased ABA content enables plants to tolerate unfavorable environments (Truong et al., 2021). ABA accumulation can speed up stomatal closure and upregulate the expression of stress-responsive genes, resulting in increasing salt stress tolerance in plants (Sah et al., 2016). In this study, changes in ABA level in wheat seedlings under salt stress by exogenous jasmonic acid were observed (Figure 5). ABA content was significantly increased in roots of wheat seedling under salt stress compared with the control. A similar increase in ABA content by salt stress were also observed in soybean (Hamayun et al., 2015), tomato (Feng et al., 2019) and barley (Torun et al., 2020). Thus, the high accumulation of ABA could help to induce stomatal closure and reduce water loss in plants. However, it was noticed that ABA content was further significantly increased in JA pretreated wheat seedlings under salt stress in comparison with salt stress alone (Figure 5). Similarly, salicylic acid (SA) pretreatment increased ABA levels by ~81% in barley under 300 mM NaCl conditions (Torun et al., 2020). Therefore, one may speculate that the genes involved in ABA biosynthesis pathways play crucial roles in stress tolerance of plants. In the present study, the expression levels of three 9-cis-epoxycarotenoid dioxygenase genes (DN40458_c0_g7, DN58559_c0_g2 and DN60068_c2_g1), which were the key genes in regulating ABA biosynthesis were up-regulated and expressed in root of JA+NaCl treatment compared to NaCl stress (Figure 4A). Our finding is in agreement with a study showing that salt stress inhibits the biosynthesis of ABA in roots and leaves and that after trehalose treatment, the biosynthesis of ABA is greatly enhanced in roots and leaves and thus improving salt tolerance in tomato plants exposed to salinity stress (Feng et al., 2019). In *Arabidopsis thaliana*, an abscisic acid (ABA) biosynthetic gene *NCED3*, which is highly upregulated in transgenic plants, enhanced the accumulation of ABA and increased salt stress tolerance (Truong et al., 2021). Likewise, the expression levels of two genes coding aldehyde oxidases 2 (DN60148_c0_g2 and DN42765_c0_g1), which catalyze the last step in ABA biosynthesis, were induced in JA pretreated wheat seedlings under salt stress. Thus, the marked increase in ABA level induced by JA, accompanied with an increase in the transcript levels of ABA biosynthesis genes, significantly contributed to the tolerance to salt stress in wheat.

JA is a novel plant growth regulator, as well as an inducible endogenous signal molecule, which participates in the defense response of plants under various stress conditions and thereby enhances plant resistance (Sheteiwy et al., 2021; Wang et al., 2021). Changes in levels of JA in plants have been recognized as an adaptive strategy of protection against abiotic stresses (Sheteiwy et al., 2021). Endogenous JA biosynthesis occurs *via* the allene oxide synthase (AOS) branch of the oxylipin metabolite pathway, including key enzymes such as linoleate lipoxygenase (LOX), allene oxide synthase (AOS), and oxophytodienoate reductase (OPR) (Wang et al., 2021). In this study, JA content of wheat seedlings accumulated significantly under salt stress, which was consistent with the results of Sheteiwy et al. (2021) in soybean and Kang et al. (2005) in rice, indicating JA directly responded to salt stress. Based on our transcriptome analysis, the transcripts of genes associated with linoleate 9S-lipoxygenase, lipoxygenase 3 (LOX3) and allene oxide synthase (AOS) were significantly up-regulated in JA treated wheat seedlings under salt stress (Figure 4B). LOX3, an enzyme involved in JA synthesis, was shown to be increasingly induced under salt stress and enhanced salt tolerance in *Arabidopsis* (Ding et al., 2016). Likewise, the content of JA in wheat seedling was significantly increased in JA treated wheat seedlings under salt stress. The result was similar to the findings of Sheteiwy et al. (2021) in soybean, who found spraying exogenous JA increased the levels of JA in soybean seedlings under salt stress by 52.25% when compared with the control. The increase of JA level can induce expression of stress-responsive genes in plants, which could improve plant tolerance under salt stress. Therefore, our results demonstrated that exogenous JA induced the expressions of JA synthesis-related genes under salt stress, subsequently increased the JA concentration in roots and thus improved salt tolerance of wheat.

SA, a phenolic hormone that is considered to be a stress hormone, plays an important role in stress tolerance of plants by mediating a broad range of physiological and metabolic responses (Kim et al., 2017; Wu et al., 2018). It is generally accepted that the pathways for the synthesis of SA in plants include the isochorismate synthase (ICS) and phenylalanine ammonia lyase (PAL) pathways, and these enzymes such as phenylalanine ammonia-lyase and isochorismate synthase, play crucial roles in these two pathways, respectively (Lefevere et al., 2020). In present study, a gene associated with phenylalanine ammonia-lyase and two genes associated isochorismate synthase in SA biosynthesis pathway were up-regulated in JA treated wheat seedlings under salt stress (Figure 4C). Furthermore, the contents of SA in wheat seedling roots were significantly increased in JA treated wheat seedlings under salt stress. Similar results were found by Kim et al. (2017), who reported that SA levels in cucumber leaves were significantly increased after salt stress, and that SA levels were further notably increased in exogenous SA-pretreated cucumber plants under stress conditions. In addition, Iqbal et al. (2006) found that CaCl_2_ pretreatment considerably increased SA concentrations in leaves of hexaploid wheat under saline conditions. Several studies have previously confirmed that the increasing contents of SA promote stomatal closure and reduce transpiration to maintain plant water balance, and thus reduce salt stress (Wu et al., 2018; Bharath et al., 2021). Thus, exogenous JA treatment markedly increased the levels of SA in wheat seedlings under salt conditions, but the regulatory mechanisms is still obscure. Further exploration is needed to clearly demonstrate the explicit mechanisms.

## Conclusion

In this study, we presented here a comprehensive study of JA mediated enhancement of salt tolerance in wheat using the biophysiochemical and RNA-Seq analysis. The morphological features, contents of hormones (i.e., SA, ABA, and JA), antioxidant system and their related genes were involved in stress tolerance in wheat. Our study provides the first evidence of the regulatory and molecular roles of JA, at the transcription level, to enhance wheat tolerance to salt stress and demonstrates the potential application of this hormone in crop breeding and growth under salt stress conditions.

## Data availability statement

The datasets presented in this study can be found in online repositories. The names of the repository/repositories and accession number(s) can be found in the article/supplementary material.

## Author contributions

MZ carried out the transcriptomics analysis and wrote up the initial manuscript. YL, XD, SS, and PC participated in the experiments, performed the statistical analysis, and prepared the figures and tables. ZQ conceived of the study and critically edited the whole manuscript. All authors read and approved the final manuscript. All authors contributed to the article and approved the submitted version.

## Funding

This research was supported by the National Natural Science Foundation of China (No.: 31500499) and the Scientific and Technological Project of Henan Province (No.: 222102110230).

## Conflict of interest

The authors declare that the research was conducted in the absence of any commercial or financial relationships that could be construed as a potential conflict of interest.

## Publisher's note

All claims expressed in this article are solely those of the authors and do not necessarily represent those of their affiliated organizations, or those of the publisher, the editors and the reviewers. Any product that may be evaluated in this article, or claim that may be made by its manufacturer, is not guaranteed or endorsed by the publisher.

## References

[B1] Ahmad P. Alyemeni M. N. Leonard W. Pravej A. Mohammad A. Saud A. A. (2017). Jasmonic acid alleviates negative impacts of cadmium stress by modifying osmolytes and antioxidants in faba bean (*Vicia faba* L.). Arch. Agron. Soil Sci. 63, 1889–1899. 10.1080/03650340.2017.1313406

[B2] Alam M. M. Nahar K. Hasanuzzaman M. Fujita M. (2014). Exogenous jasmonic acid modulates the physiology, antioxidant defense and glyoxalase systems in imparting drought stress tolerance in different Brassica species. Plant Biotechnol. Rep. 8, 279–293. 10.1007/s11816-014-0321-8

[B3] Al-Zahrani H. S. Alharby H. F. Fahad S. (2022). Antioxidative defense system, hormones, and metabolite accumulation in different plant parts of two contrasting rice cultivars as influenced by plant growth regulators under heat stress. Front. Plant Sci. 13, 911846. 10.3389/fpls.2022.911846 35712584 PMC9196032

[B4] Barrs H. D. Weatherley P. E. (1962). A re-examination of the relative turgidity technique for estimating water deficits in leaves. Aust. J. Biol. Sci. 15, 413–428. 10.1071/BI9620413

[B5] Benjamini Y. Yekutieli D. (2001). The control of the false discovery rate in multiple testing under dependency. Ann. Stat. 29, 1165–1188. 10.1214/aos/101369999818298808

[B6] Bharath P. Gahir S. Raghavendra A. S. (2021). Abscisic acid-induced stomatal closure: an important component of plant defense against abiotic and biotic stress. Front. Plant Sci. 12, 324. 10.3389/fpls.2021.615114 33746999 PMC7969522

[B7] Conesa A. Gotz S. Garcia-Gomez J. M. Terol J. Talon M. Robles M. (2005). Blast2go: a universal tool for annotation, visualization and analysis in functional genomics research. Bioinformatics 21, 3674–3676. 10.1093/bioinformatics/bti610 16081474

[B8] Ding H. Lai J. Wu Q. Zhang S. Chen L. Dai Y. S.. (2016). Jasmonate complements the function of Arabidopsis lipoxygenase 3 in salinity stress response. Plant Sci. 244, 1–7 10.1016/j.plantsci.2015.11.009 26810448

[B9] Fahad S. Hussain S. Matloob A. Khan F. A. Khaliq A. Saud S.. (2014). Phytohormones and plant responses to salinity stress: a review. Plant Growth Regul. 75, 391–404. 10.1007/s10725-014-0013-y

[B10] Fahad S. Hussain S. Saud S. Hassan S. Ihsan Z. Shah A. N.. (2016). Exogenously applied plant growth regulators enhance the morphophysiological growth and yield of rice under high temperature. Front. Plant Sci. 7, 1250. 10.3389/fpls.2016.01250 27625658 PMC5003834

[B11] Farooq M. A. Gill R. A. Islam F. Ali B. Liu H. Xu J.. (2016). Methyl jasmonate regulates antioxidant defense and suppresses arsenic uptake in *Brassica napus* L. Front. Plant Sci. 7, 468. 10.3389/fpls.2016.00468 27148299 PMC4826882

[B12] Feng Y. Chen X. He Y. Kou X. Xue Z. (2019). Effects of exogenous trehalose on the metabolism of sugar and abscisic acid in tomato seedlings under salt stress. Trans. Tianjin Univ. 25, 451–471. 10.1007/s12209-019-00214-x

[B13] Formentin E. Sudiro C. Perin G. Riccadonna S. Barizza E. Baldoni E.. (2018). Transcriptome and cell physiological analyses in different rice cultivars provide new insights into adaptive and salinity stress responses. Front. Plant Sci. 9, 204. 10.3389/fpls.2018.00204 29556243 PMC5844958

[B14] Ghassemi-Golezani K.. (2018). Foliar sprays of salicylic acid and jasmonic acid stimulate H^+^-ATPase activity of tonoplast, nutrient uptake and salt tolerance of soybean. Ecotox. Environ. Saf. 166, 18–25. 10.1016/j.ecoenv.2018.09.059 30240931

[B15] Giannopolitis C. N. Ries S. K. (1977). Superoxide dismutases: I. occurrence in higher plants. Plant Physiol. 59, 309–314. 10.1104/pp.59.2.309 16659839 PMC542387

[B16] Grabherr M. G. Haas B. J. Yassour M. Levin J. Z. Thomson D. A. Amit I.. (2011). Full-length transcriptome assembly from RNA-Seq data without a reference genome. Nat. Biotechnol. 29, 644–652. 10.1038/nbt.1883 21572440 PMC3571712

[B17] Hamayun M. Hussain A. Khan S. A. Irshad M. Khan A. L. Waqas M.. (2015). Kinetin modulates physio-hormonal attributes and isoflavone contents of soybean grown under salinity stress. Front. Plant Sci. 6, 377. 10.3389/fpls.2015.00377 26082785 PMC4450176

[B18] Hong Y. Zhang H. Huang L. Li D. Song F. (2016). Overexpression of a stress-responsive NAC transcription factor gene O*NAC022* improves drought and salt tolerance in rice. Front. Plant Sci. 7, 403. 10.3389/fpls.2016.00004 26834774 PMC4722120

[B19] Huang Q. Farooq M. A. Hannan F. Chen W. Ayyaz A. Zhang K.. (2022). Endogenous nitric oxide contributes to chloride and sulphate salinity tolerance by modulation of ion transporter expression and reestablishment of redox balance in Brassica napus cultivars. Environ. Exp. Bot. 194, 104734. 10.1016/j.envexpbot.2021.104734

[B20] Huang Q. Wang Y. Li B. Chang J. Chen M. Li K. (2015). *TaNAC29*, a NAC transcription factor from wheat, enhances salt and drought tolerance in transgenic *Arabidopsis*. BMC Plant Biol. 15, 268. 10.1186/s12870-015-0644-9 26536863 PMC4632686

[B21] Iqbal M. Ashraf M. Jamil A. Rehman S. (2006). Does seed priming induce changes in the levels of some endogenous plant hormones in hexaploid wheat plants under salt stress. J. Integr. Plant Biol. 48, 181–189. 10.1111/j.1744-7909.2006.00181.x

[B22] Kang D. J. Seo Y. J. Lee J. D. Ishii R. Kim K. U. Shin D. H. (2005). Jasmonic acid differentially affects growth, ion uptake and abscisic acid concentration in salt-tolerant and salt-sensitive rice cultivars. J. Agron. Crop Sci. 191, 273–282. 10.1111/j.1439-037X.2005.00153.x

[B23] Kim Y. Kim S. Shim I. S. (2017). Exogenous salicylic acid alleviates salt stress damage in cucumber under moderate nitrogen conditions by controlling endogenous salicylic acid levels. Hortic. Environ. Biotechnol. 58, 247–253. 10.1007/s13580-017-0111-7

[B24] Lefevere H. Bauters L. Gheysen G. (2020). Salicylic acid biosynthesis in plants. Front. Plant Sci. 11, 338. 10.3389/fpls.2020.00338 32362901 PMC7182001

[B25] Liu Q. Tang J. Wang W. Zhang Y. Yuan H. Huang S. (2018). Transcriptome analysis reveals complex response of the medicinal/ornamental halophyte *Iris halophila* Pall. to high environmental salinity. Ecotox. Environ. Saf. 15, 250–260. 10.1016/j.ecoenv.2018.09.003 30199796

[B26] Livak K. J. Schmittgen T. D. (2001). Analysis of relative gene expression data using real-time quantitative PCR and the 2^−ΔΔCt^ method. Methods 25, 402–408 10.1006/meth.2001.1262 11846609

[B27] Ma J. Li R. Wang H. Li D. Wang X. Zhang Y.. (2017). Transcriptomics analyses reveal wheat responses to drought stress during reproductive stages under field conditions. Front. Plant Sci. 8, 592. 10.3389/fpls.2017.00592 28484474 PMC5399029

[B28] Ma Q. Xia Z. Cai Z. Li L. Cheng Y. Liu J.. (2019). *GmWRKY16* enhances drought and salt tolerance through an ABA-mediated pathway in *Arabidopsis thaliana*. Front. Plant Sci. 9, 1979. 10.3389/fpls.2018.01979 30740122 PMC6357947

[B29] Mao X. Zhang H. Qian X. Li A. Zhao G. Jing R. (2012). *TaNAC2*, a NAC-type wheat transcription factor conferring enhanced multiple abiotic stress tolerances in *Arabidopsis*. J. Exp. Bot. 63, 2933–2946. 10.1093/jxb/err462 22330896 PMC3350912

[B30] Nahhas N. E. AlKahtani M. F. Abdelaal K. A. Husnain L. A. AlGwaiz H. M. Hafez Y. M.. (2021). Biochar and jasmonic acid application attenuates antioxidative systems and improves growth, physiology, nutrient uptake and productivity of faba bean (*Vicia faba* L.) irrigated with saline water. Plant Physiol. Biochem. 166, 807–817. 10.1016/j.plaphy.2021.06.033 34225005

[B31] Nakano Y. Asada K. (1981). Hydrogen peroxide is scavenged by ascorbate-specific peroxidase in spinach chloroplasts. Plant Cell Physiol. 22, 867–880.

[B32] Qiu Z. B. Guo J. L. Zhu A. J. Zhang L. Zhang M. M. (2014). Exogenous jasmonic acid can enhance tolerance of wheat seedlings to salt stress. Ecotox. Environ. Safe. 104, 202–208. 10.1016/j.ecoenv.2014.03.014 24726929

[B33] Qiu Z. B. Hai B. Z. Li Y. F. Guo J. L. Zhang L. (2016). Characterization of wheat miRNAs and their target genes responsive to cadmium stress. Plant Physiol. Biochem. 101, 60–67 10.1016/j.plaphy.2016.01.020 26854408

[B34] Qiu Z. B. Yuan M. He Y. Li Y. Zhang L. (2017). Physiological and transcriptome analysis of He-Ne laser pretreated wheat seedlings in response to drought stress. Sci. Rep. 7, 6108 10.1038/s41598-017-06518-z 28733678 PMC5522386

[B35] Réthoré E. Ali N. Yvin J. C. Hosseini S. A. (2020). Silicon regulates source to sink metabolic homeostasis and promotes growth of rice plants under sulfur deficiency. Int. J. Mol. Sci. 21, 3677–3698. 10.3390/ijms21103677 32456188 PMC7279143

[B36] Sah S. K. Reddy K. R. Li J. (2016). Abscisic acid and abiotic stress tolerance in crop plants. Front. Plant Sci. 7, 571. 10.3389/fpls.2016.00571 27200044 PMC4855980

[B37] Shen N. Wang T. Gan Q. Liu S. Wang L. Jin B. (2022). Plant flavonoids: classification, distribution, biosynthesis, and antioxidant activity. Food Chem. 383,132531. 10.1016/j.foodchem.2022.132531 35413752

[B38] Sheteiwy M. S. Shao H. Qi W. Daly P. Sharma A. Shaghaleh H.. (2021). Seed priming and foliar application with jasmonic acid enhance salinity stress tolerance of soybean (*Glycine max* L.) seedlings. J. Sci. Food Agric. 101, 2027–2041. 10.1002/jsfa.10822 32949013

[B39] Sirhindi G. Mir M. A. Abd-Allah E. F. Ahmad P. Gucel S. (2016). Jasmonic acid modulates the physio-biochemical attributes, antioxidant enzyme activity, and gene expression in *Glycine max* under nickel toxicity. Front. Plant Sci. 7, 591. 10.3389/fpls.2016.00591 27242811 PMC4864666

[B40] Torun H. Novák O. Mikulík J. Pěnčík A. Strnad M. Ayaz F. A. (2020). Timing-dependent effects of salicylic acid treatment on phytohormonal changes, ROS regulation, and antioxidant defense in salinized barley (*Hordeum vulgare* L.). Sci. Rep. 10, 13886. 10.1038/s41598-020-70807-3 32807910 PMC7431421

[B41] Truong H. A. Lee S. Trinh C. S. Lee W. J. Chung E. H. Hong S. W.. (2021). Overexpression of the HDA15 gene confers resistance to salt stress by the induction of NCED3, an ABA biosynthesis enzyme. Front. Plant Sci. 12, 640443. 10.3389/fpls.2021.640443 33995439 PMC8120240

[B42] Wang C. Deng P. Chen L. Wang X. Ma H. Hu W.. (2013). A wheat WRKY transcription factor *TaWRKY10* confers tolerance to multiple abiotic stresses in transgenic tobacco. PLoS ONE 8, e65120. 10.1371/journal.pone.0065120 23762295 PMC3677898

[B43] Wang M. Zhang X. Liu J. H. (2015). Deep sequencing-based characterization of transcriptome of trifoliate orange (*Poncirus trifoliata* (L.) Raf.) in response to cold stress. BMC Genomics 16, 555. 10.1186/s12864-015-1629-7 26219960 PMC4518522

[B44] Wang Y. Mostafa S. Zeng W. Jin B. (2021). Function and mechanism of jasmonic acid in plant responses to abiotic and biotic stresses. Int. J. Mol. Sci. 22, 8568. 10.3390/ijms22168568 34445272 PMC8395333

[B45] Wei Q. Luo Q. Wang R. Zhang F. He Y. Zhang Y.. (2017). A wheat R2R3-type MYB transcription factor *TaODORANT1* positively regulates drought and salt stress responses in transgenic tobacco plants. Front. Plant Sci. 8, 1374. 10.3389/fpls.2017.01374 28848578 PMC5550715

[B46] Wu L. Huang Z. Li X. Ma L. Gu Q. Wu H.. (2018). Stomatal closure and SA-, JA/ET-signaling pathways are essential for *Bacillus amyloliquefaciens* FZB42 to restrict leaf disease caused by *Phytophthora nicotianae* in *Nicotiana benthamiana*. Front. Microbiol. 9, 847. 10.3389/fmicb.2018.00847 29755447 PMC5934478

[B47] Xu Y. Huang B. (2018). Comparative transcriptomic analysis reveals common molecular factors responsive to heat and drought stress in *Agrostis stolonifera*. Sci. Rep. 8, 15181. 10.1038/s41598-018-33597-3 30315246 PMC6185948

[B48] Xu Z. Wang C. Xue F. Zhang H. Ji W. (2015). Wheat NAC transcription factor *TaNAC29* is involved in response to salt stress. Plant Physiol. Biochem. 96, 356–363. 10.1016/j.plaphy.2015.08.013 26352804

[B49] Yao L. Wang J. Li B. Meng Y. Ma X. Si E.. (2018). Transcriptome sequencing and comparative analysis of differentially-expressed isoforms in the roots of *Halogeton glomeratus* under salt stress. Gene 646, 159–168. 10.1016/j.gene.2017.12.058 29292193

[B50] Zhang J. Kirkham M. (1994). Drought-stress-induced changes in activities of superoxide dismutase, catalase and peroxidase in wheat species. Plant Cell Physiol. 35, 785–791. 10.1093/oxfordjournals.pcp.a078658

[B51] Zhang Y. Fan Y. Rui C. Zhang H. Xu N. Dai M.. (2021). Melatonin improves cotton salt tolerance by regulating ROS scavenging system and Ca^2+^ signal transduction. Front. Plant Sci. 12, 693690. 10.3389/fpls.2021.693690 34262587 PMC8273866

[B52] Zhou Y. Yang P. Cui F. Zhan F. Luo X. Xie J. (2016). Transcriptome analysis of salt stress responsiveness in the seedlings of Dongxiang wild rice (*Oryza rufipogon* Griff.). PLOS ONE 11, e0146242. 10.1371/journal.pone.0146242 26752408 PMC4709063

[B53] Zhu M. Duan X. Cai P. Li Y. F. Qiu Z. B. (2022c). Deciphering the genome of *Simplicillium aogashimaense* to understand its mechanisms against the wheat powdery mildew fungus *Blumeria graminis* f. sp. tritici. Phytopathol. Res. 4, 16. 10.1186/s42483-022-00121-5

[B54] Zhu M. Duan X. Zeng Q. Q. Cai P. Shi W. Qiu Z. B. (2022a). Podosphaera xanthii causing powdery mildew on Impatiens balsamina in China. Can. J. Plant Pathol. 44, 354–360 10.1080/07060661.2021.2011421

[B55] Zhu M. Duan X. Zeng Q. Q. Liu Y. Qiu Z. B. (2022b). He-Ne laser irradiation ameliorates cadmium toxicity in wheat by modulating cadmium accumulation, nutrient uptake and antioxidant defense system. Ecotox. Environ. Saf. 236, 113477. 10.1016/j.ecoenv.2022.113477 35367883

