# Supplementary material for: Jasmonic acid pretreatment improves salt tolerance of wheat by regulating hormones biosynthesis and antioxidant capacity
Source: Front Plant Sci. 2022 Jul 22;13:968477. doi: 10.3389/fpls.2022.968477 (PMC9355640; doi:10.3389/fpls.2022.968477)
Supplement: Supplementary file 1 [file Data_Sheet_1.doc]

**Table S1 HPLC-ESI-MS/MS conditions used for analysis and quantification**

**of the SA, JA and ABA**

| Equipment | Agilent 1290 HPLC, SCIEX 6500 Q-TRAP |
| --- | --- |
| Column | Poroshell 120 SB-C18 column (2.1mm×150mm, 2.7 µm) |
| Curtain gas | 15 psi |
| Nebulizer gas | 65 psi |
| Heater gas | 70 psi |
| Injector temperature | 400°C |
| Ion source voltage  m/z | 4500 v  SA (137, 92.9); JA (209.2, 58.9); ABA (263.1, 153) |

| **Gene name** | **Gene description** | **Forward sequences (5'-3')** | **Reverse sequences (5'-3')** |
| --- | --- | --- | --- |
| Tublulin_DQ435660.1 | Internal control | CCGTGGTGATGTTGTGCCAAAGGA | CGACGACACTGGTGGAGTTGGAGA |
| TRINITY_DN51814_c0_g8 | Gibberellin 20 oxidase 1 | GCCAGTTCCGCATCAA | ACGCAGTCGTCCTCCT |
| TRINITY_DN53724_c1_g2 | Glutathione S-transferase | TGCCCGTCCTCATTCACAA | TGCCTCAAACCAGGGAAGATA |
| TRINITY_DN59872_c1_g1 | Catalase | ATTCCACTCAACTACAGGCACA | CCTCCTACAGTAACGGCTTCAT |
| TRINITY_DN39447_c1_g1 | Copper chaperone for superoxide dismutase | CCTCCTCGTCCTCCTCTTCTC | AAATCATCTGGGTTCCCTTGC |
| TRINITY_DN43080_c3_g2 | Drought and salt tolerance protein | AACAACGGCGAGGATG | AAGGGCGGTAGGGGAC |
| TRINITY_DN57819_c0_g1 | Peroxidase 21 | TCGTGAAGGAGCAGGTGAGGAG | TGTCGTTGTGGTTCGGGATGTA |
| TRINITY_DN54646_c1_g2 | WRKY transcription factor | GTGCCCAAGTTCAAGTCCG | TCGCCTGCCAGTCGTATCT |
| TRINITY_DN45919_c0_g1 | MYB transcription factor | TTGCTGCGAGAAGATGG | GGGCGGAGGTAGTTGAT |
| TRINITY_DN49382_c0_g2 | NAC Transcription factor | CTGCTGGCGTAGTAGGGA | GGTGAAGAAGGAGGAGGC |
| TRINITY_DN44912_c0_g1 | R2R3-type MYB transcription factor | CCTCCTTCAGCGGCATCAC | TCATCGCCATCGCCACCA |
| TRINITY_DN48029_c1_g4 | chlorophyll a-b binding protein | CTACGCCACCACCTCC | GACTCCTTGAACCGCT |
| TRINITY_DN47172_c2_g1 | Leucine-rich repeat receptor-like protein kinase | CTATGTCACCTGCGATGC | ATGTTGTTGCTGGAGATGTT |

**Table S2** Primers used for qRT-PCR analysis

**Table S3** Length distribution of assembled transcripts and unigenes

| Nucleotide length | Transcripts | Unigene |
| --- | --- | --- |
| 200-500 | 74,598 | 34,150 |
| 500-1000 | 37,645 | 10,032 |
| 1000-1500 | 17,966 | 5,321 |
| 1500-2000 | 7,595 | 2,776 |
| >=2000 | 4,436 | 1,984 |
| Min length | 201 | 201 |
| Median length | 470 | 343 |
| Max length | 10,795 | 10,795 |
| N50(bp) | 965 | 985 |

**Table S4** Functional annotation of *T. aestivum* unigenes against the public databases

|  | Number of unigenes | Percentage (%) |
| --- | --- | --- |
| All | 54,263 | 100.00 |
| GO | 16,596 | 30.58 |
| KEGG | 9,124 | 16.81 |
| Pfam | 15,874 | 29.25 |
| Swiss-Prot | 13,811 | 25.45 |
| COG | 21,403 | 39.44 |
| Nr | 24,388 | 44.94 |
|  |  |  |


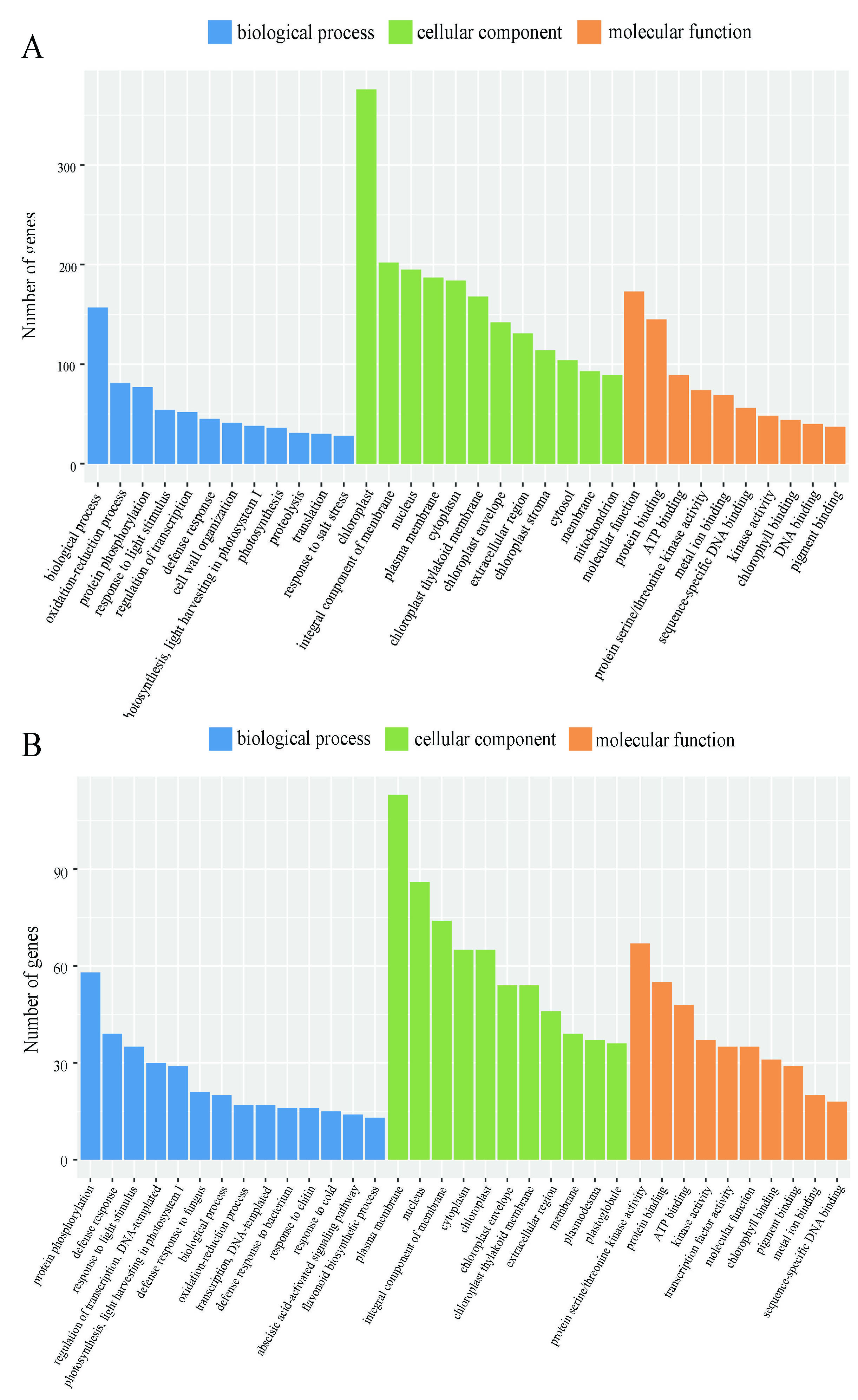
 **Fig.S1.** GO term enrichment analysis of DEGs in JA pretreated wheat seedlings under NaCl stress for 3 days. A NaCl vs CK B JA+NaCl vs NaCl.
